# Subarachnoid-Pleural Fistula: Applied Anatomy of the Thoracic Spinal Nerve Root

**DOI:** 10.5402/2011/168959

**Published:** 2011-09-04

**Authors:** Mohammed F. Shamji, Sudhir Sundaresan, Vasco Da Silva, Jean Seely, Farid M. Shamji

**Affiliations:** ^1^Division of Neurosurgery, The Ottawa Hospital, Ottawa, ON, Canada K1Y 4E9; ^2^Division of Thoracic Surgery, The Ottawa Hospital, Ottawa, ON, Canada K1H 8L6; ^3^Department of Radiological Sciences, The Ottawa Hospital, Ottawa, ON, Canada K1H 8L6

## Abstract

Subarachnoid-pleural fistula (SPF) is a rare complication of chest or spine operations for neoplastic disease. Concomitant dural and parietal pleural defects permit flow of cerebrospinal fluid into the pleural cavity or intrapleural air into the subarachnoid space. Dural injury recognized intraoperatively permits immediate repair, but unnoticed damage may cause postoperative pleural effusion, intracranial hypotension, meningitis, or pneumocephalus. We review two cases of SPF following surgical intervention for chest wall metastatic disease to motivate a detailed review of the anatomy of neural, osseous, and ligamentous structures at the intervertebral foramen. We further provide recommendations for avoidance and detection of such complication.

## 1. Introduction

Subarachnoid-pleural fistula (SPF) is an abnormal communication between the subarachnoid and pleural spaces that normally can arise from blunt or penetrating trauma or as a complication of chest operation. This occurs infrequently following cardiothoracic surgery for lung or chest wall [1] resection and closure of patent ductus arteriosus [2], spine surgery for transthoracic discectomy [3, 4], removal of vertebral tumors [5], or spinal fusion [6]. Symptoms develop from the accumulation of pleural fluid, the reduced volume of cerebrospinal fluid (CSF), the presence of intracranial air, or the development of meningitis, with outcomes ranging from benign to catastrophic. This complication most frequently occurs in the setting of a tumour that occupies the costovertebral angle requiring extrapleural dissection, excessive rib retraction, or disarticulation of the costotransverse joint leading to nerve root avulsion or dural tear. The risk is increased when there is associated inadvertent posterior rib neck fracture.

Early recognition of SPF is important for timely repair because spontaneous closure seldom occurs. Following confirmation of the SPF diagnosis by computed tomography (CT) myelography or radionuclide cisternography, conservative therapies such as lumbar drainage to divert CSF flow may be successful, though this is less likely to occur in patients who have undergone previous surgery or received radiotherapy to the affected area. Consequently, direct operative repair of the dural defect with fine non-absorbable suture and reinforcing repair with a tissue patch is often necessary for permanent definitive treatment of the established SPF [5]. 

This paper describes the diagnosis and management of two cases of SPF encountered by the senior author that illustrate the typical course to presentations of this complication. This motivates a detailed anatomic description of cerebrospinal fluid fistulas to the pleural space, with elaboration of intraoperative techniques to lessen the likelihood of such complication. 

## 2. Case Reports

### 2.1. Case Report 1

An 18-year-old woman with a known diagnosis of Wilm's tumor had undergone left nephrectomy and adjuvant chemotherapy at 15 months of age. Tumor recurrence was noted six months later in both lungs for which she received additional chemotherapy and bilateral lung radiotherapy. Between ages 15 and 17 years, she experienced several bouts of recurrent disease in the right chest treated by stem cell transplant, chemotherapy, and radiotherapy. A severe septic complication developing after the stem cell transplant required 12 weeks of intensive care.

**Figure 1:**
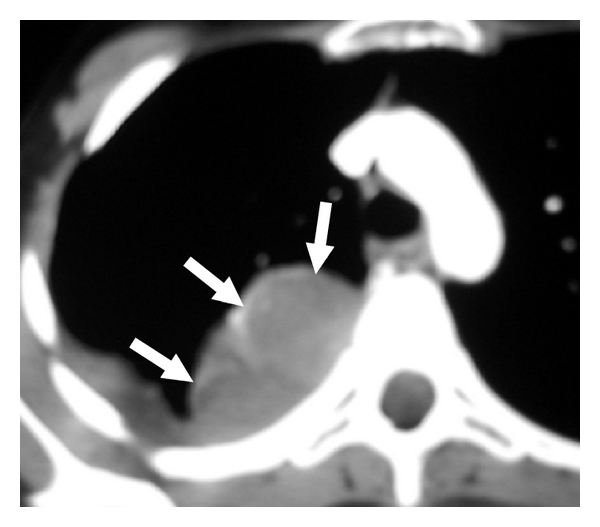
Axial contrast-enhanced CT of a 17-year-old girl demonstrates a heterogeneous soft tissue mass in the right hemithorax (arrows) that abuts the costal and vertebral pleura, with no frank evidence of bony invasion. A small right pleural effusion is also present. There is no visible tumor invasion into the spinal canal. Biopsy of the lesion confirmed metastatic Wilm's tumor.

Thoracic Surgery service was consulted for management of disabling persistent right posterior chest wall pain due to multiple pleural-based tumor recurrence in the paravertebral gutter and lung parenchyma (Figure 1). The goals of surgical treatment were disease control and palliation. Right thoracotomy was performed, and complete resection was carried out by extrapleural and intrapericardial pneumonectomy. Intraoperatively, a CSF leak was recognized and treated with a free muscle plug patch inserted in the aperture of the intervertebral foramen and reinforced with Tissel glue (Baxter Biosurgery, Deerfield, Il).

**Figure 2:**
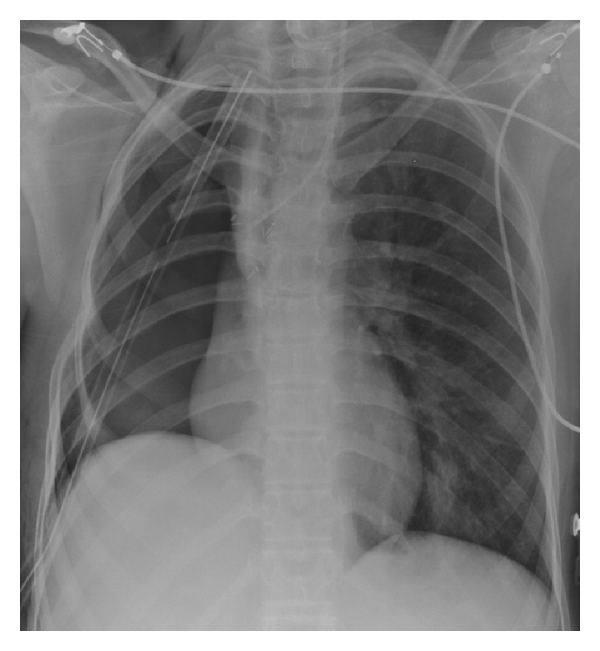
Supine portable chest radiograph obtained 12 hours after resection of the Wilm's metastasis shows the expected early postoperative appearance of the right pneumonectomy space, thoracotomy and bony resection of the 5th right posterior rib, midline position of the mediastinum, and predominantly air in the pneumonectomy space.

**Figure 3:**
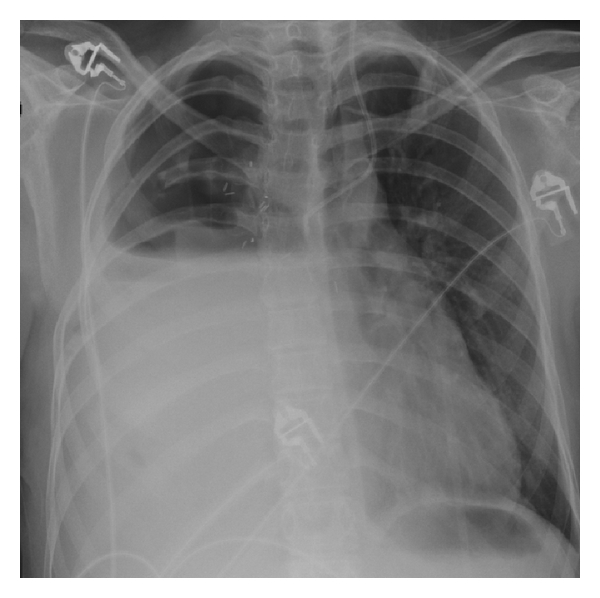
Upright portable chest radiograph obtained 9 days after surgery demonstrates rapid fluid accumulation in the right pneumonectomy space with contralateral shift of mediastinal structures to the left. Both findings are suggestive of excessive fluid volume in the pneumonectomy space.

**Figure 4:**
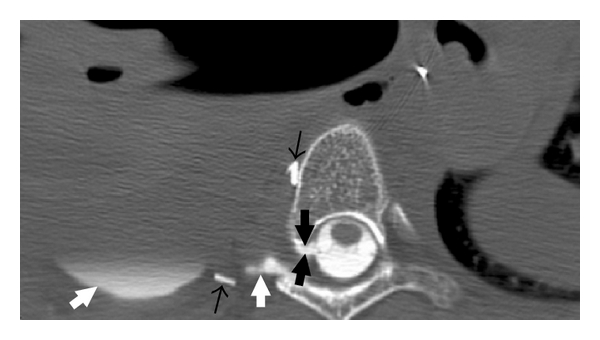
Axial CT obtained after contrast myelogram demonstrates the subarachnoid-pleural fistula (thick black arrows) establishing communication between the spinal canal and the right pleural effusion. Contrast is observed to layer dependently in the right pleural space (white arrows). Note the adjacent surgical clips (thin black arrows).

**Figure 5:**
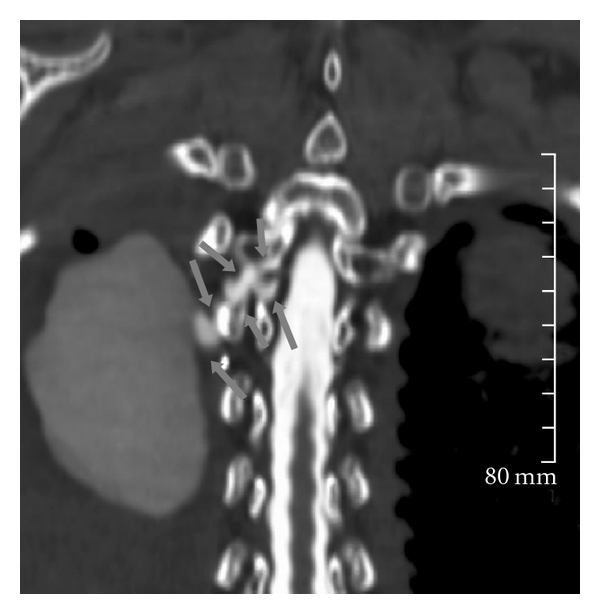
Coronal reformatted CT after contrast myelogram shows the subarachnoid pleural fistula (arrows) connecting the radio-opaque subarachnoid space with the large area of dilute contrast in the right pleural space.

In the early postoperative period, serial chest radiographs failed to demonstrate the expected mediastinal shift to the right hemithorax (Figures 2 and 3). This prompted early investigation with a CT myelogram that confirmed a suspected SPF at the right T4/5 intervertebral foramen (Figures 4 and 5). She was immediately referred for definitive neurosurgical care involving unilateral T4 and T5 laminectomy that revealed CSF leaking from an avulsed nerve root on the lateral aspect of the dural sac. A nonabsorbable, 6-0 prolene, continuous suture from caudal to cephalad was used to close this 7 mm defect. The integrity of the repair was confirmed by a Valsalva maneuver during which no CSF was observed to leak. The suture line was reinforced with a 2 mm layer of fibrin sealant.

The patient's postoperative course was significant only for the development of pneumonia for which she received in-patient treatment. At followup 8 months later, there was no clinical or radiographic evidence of persistent or recurrent SPF.

### 2.2. Case Report 2

**Figure 6:**
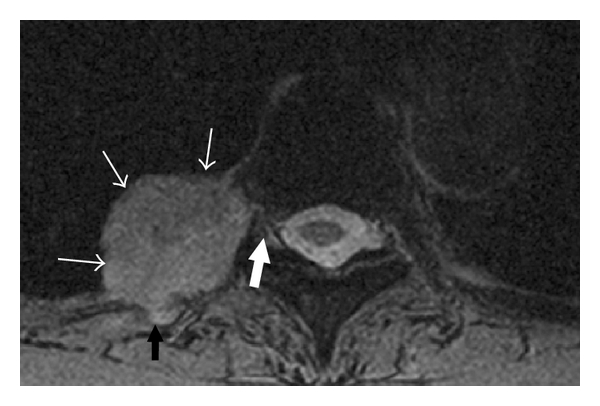
Axial T2-weighted MRI in a 66-year-old male through the upper right hemithorax demonstrates a soft tissue mass (white arrows) in the right pleural space. The lesion is observed to invade the chest wall and adjacent rib (black arrow). Note the proximity of the mass to the intervertebral foramen and spinal canal (thick white arrow).

A 66-year-old man with chronic liver disease due to Hepatitis B developed hepatocellular carcinoma for which a right partial hepatectomy was performed. Two years later, a solitary 7 mm metastasis was removed from the lower lobe of the right lung by video-assisted thoracoscopic surgery wedge resection. Two years after that, further recurrence was noted in the right lung and in the adjoining parietal pleura at the site of the previous resection. These were removed by wedge resection using open technique at which time concomitant complete parietal pleurectomy was required to remove several other small remote tumor nodules. One year later, tumour recurrence was again noted in the right hemithorax in the para-vertebral gutter. This lesion in the posterior segment of the lower lobe measured 3 cm in size and involved the adjoining 5th rib in close proximity to the intervertebral foramen (Figure 6). Palliative and prophylactic resection was recommended to relieve persistent chest wall pain and because of the potential of tumor extending centrally into the intervertebral foramen with consequent epidural spinal cord compression or meningeal invasion with CSF carcinomatosis. Wide en bloc resection was performed with removal of the involved posterior portion of the 5th rib, the T5 transverse process, the adjoining cortex of T5 vertebral body, and the posterior segment of the upper lobe with the necessary extrapleural dissection. During that dissection, a CSF leak was observed at the 5th intercostal nerve, with control achieved by application of two large hemoclips after which the nerve was divided. A Valsalva maneuver was then performed during which no further CSF was observed to leak. The postoperative course was uneventful.

## 3. Discussion

A rare complication of chest operation, particularly in the paravertebral gutter region, is the development of fistula between the subarachnoid and pleural spaces resulting from iatrogenic concomitant dural and pleural defects. It is reported to occur in fewer than 1% of patients undergoing en bloc lung resection with the adjoining chest wall [1], with common anatomic features being involvement of the upper lobe of the lung with chest wall invasion into the region of the costovertebral angle. Furthermore, the necrotizing potential of preoperative or intraoperative radiotherapy can impede wound healing of these defects, predisposing patients to fistula formation [7]. The patients presented in this report had chest wall involvement of Wilm's tumor and hepatocellular carcinoma. The first patient had predisposing features of preoperative radiotherapy and an identified intraoperative CSF leak. The second patient had extensive chest wall pathology in close proximity to the T5 intervertebral foramen, again with intraoperative observation of CSF leakage.

Requisite for formation of an SPF is breach of the dura, arachnoid, and parietal pleura. Violation of these membranes can more commonly lead to extradural flow of CSF or less commonly intradural accumulation of air with consequent pulmonary and neurological symptomatology. An understanding of the regional anatomy and pathophysiology that underlie the development of SPF after thoracic surgical procedures can identify sites that are vulnerable to injury during the dissection and key steps that may be taken to protect against this complication and to facilitate secondary surgical remedy when indicated.

### 3.1. Topography and Trajectory of Thoracic Spinal Nerves

The spinal nerve roots are multivesicular structures that are surrounded by merged piaarachnoid enclosure as they emerge through a dural fenestration at each segmental level [8]. The 31 paired spinal nerves, of which 12 are thoracic, are formed by the union of dorsal and ventral roots that carry afferent (sensory) and efferent (motor) fibers respectively. More commonly the dorsal and ventral roots exit through discrete fenestrations in the dura, but an anatomic variant can include the simultaneous emergence of both. The arachnoid also forms a separate sheath for both roots, with the subarachnoid space extending to the proximal dorsal root ganglion providing an anatomical route of CSF absorption into arachnoid villi [9–11]. These dural sheaths blend with the epineurium that adheres to the periosteal lining of the lateral intervertebral foramen. The dorsal root ganglion is a collection of sensory cell soma that extends processes centrally to the medulla spinalis and peripherally to the spinal nerve. It normally lies in the medial intervertebral foramen, with the confluence of the dorsal and ventral roots lying just distal to this point. It normally lies just lateral to the edge of the dural reflection, but it may be intradural. Ebraheim and coworkers [12] have quantified the anatomic relationship between the thoracic pedicle and the nerve root. Among 15 cadavers, they demonstrated that the superoinferior diameter of the nerve root increased from 2.9 mm at T1 to 4.6 mm at T12. No epidural space was seen between the dural sac and the pedicle, and the frontal angle decreased from 120.1° at T1 to 57.1° at T12. Similar work by Ugur and coworkers [13] demonstrates a decrease in mean root exit angle from 104° at T1 to 60° at T12 with nerve root diameters that increased from between 2.3 mm at T1 to T5 to 3.7 mm at T12. The junction of the two roots forms a very short segment mixed spinal nerve that divides into a large ventral and small dorsal ramus, the former of which becomes the intercostal nerve.

### 3.2. Articulations of the Thoracic Spine

The superior and inferior surfaces of adjacent vertebrae are covered with a thin layer of hyaline cartilage and united by a thick fibrocartilaginous intervertebral disc. The cartilaginous endplate covers the cortical vertebral body surfaces and permits longitudinal diffusion of nutrients to the avascular center of the disc. The outer surface of the AF is innervated, and peripheral blood vessels also penetrate radially to supply the center of the disc. The central nucleus pulposus is an avascular, viscoelastic gel with predominantly type II collagen and hydrated proteoglycans. It is normally constrained from cephalocaudal herniation by the endplates and from radial herniation by the competent annulus fibrosus. Of more surgical relevance, the anatomy of the costovertebral, costotransverse, and thoracic facet joints must be defined because of their proximity to neural structures of interest.

**(a) Costovertebral Joint**

The rib head has a facet that articulates in planar joints with a single costal facet on T1, T10, T11, and T12, with all other levels having articulations with the costal facet of their numbered level and additionally the inferior costal demifacet of the vertebral body one level above and with the associated intervertebral disc [14]. The vertebral body costal facets are found dorsally near the root of the pedicle, with more rostral location in the upper thoracic spine and more caudal location in the lower thoracic spine [15]. The ribs that have two articulations have two rib head facets with an intervening interarticular crest that has ligamentous attachments to the intervertebral disc. The capsular, radiate, and intraarticular ligaments are associated with this joint. The superior fibers of the joint capsule extend through the intervertebral foramen to attach to the posterior aspect of the intervertebral disc. The posterior capsular fibers are continuous with the fibers of the costotransverse ligament. The radiate ligaments fan out from the anterior aspect of the rib to the anterior vertebral body—superiorly to the vertebra above, laterally to the intervertebral disc, and inferiorly to the corresponding vertebra. Costovertebral joints where the rib articulates with two vertebrae also have an extrasynovial, intra-articular ligament that attaches from the crest on the rib head that lies between the two costal demifacets to the intervertebral disc.

**(b) Costotransverse Joint**

An articular region on the tubercle of the first ten ribs articulates with a facet on the corresponding transverse process [14]. These synovial joints transition from a convex rib portion in the upper thoracic spine to planar in the lower thoracic spine. The costotransverse, superior costotransverse, and lateral costotransverse ligaments are associated with this joint. The costotransverse ligament attaches the rib to the transverse process, occupying the costotransverse foramen. The superior costotransverse ligament has anterior and posterior layers—the anterior layer attaches the crest of the rib neck to the transverse process and is continuous laterally with the internal intercostal membrane, crossed by the intercostal vessels and nerves. The posterior layer attaches the dorsal aspect of the rib to the transverse process and is continuous laterally with the external intercostal muscle.

**(c) Facet Joint**

Thoracic facet joints consist of apposed articular processes lined with hyaline cartilage extending from adjacent vertebrae [14]. They are synovial joints encased in a fibrous capsule that attaches peripheral to the articular surfaces, with the superior articular facets being slightly convex, oriented 60° from the horizontal plane and 20° from the frontal plane facing posteriorly and laterally [15, 16]. The inferior facets are appropriately positioned facing anteriorly and medially to match their partner.

### 3.3. Anatomy of the Intervertebral Foramen

The thoracic spinal nerve root traverses the radicular canal from the spinal cord to the intervertebral foramen. It is divided into the retrodiscal, parapedicular, and foraminal sections, with the latter of greatest interest to the thoracic surgeon. The borders of the intervertebral foramen include the posterior vertebral body, the intervertebral disc, the lateral posterior longitudinal ligament, and the anterior longitudinal venous plexus (ventral), the superior and inferior articular processes with capsule that merges with ligamentum flavum (dorsal), and the pedicles (cranial and caudal). Approaches to the lateral thoracic spine are limited by the rib head that can create a false foramen and obscure the view of the exiting neural elements. The rib head and its facet must be removed to provide visibility to the true intervertebral foramen, to provide access to the pedicle and spinal cord, and to create a suitably flat surface for instrumentation [17]. The foramen are of variable shape: oval (26.6%), auricular (58%), or teardrop (17.4%) and are incompletely filled by the nerve root that are generally apposed to the upper pedicle [17]. The foramen are covered by the fascia that with two distinct perforations for the nerve root and intervertebral vessels, respectively. The trajectory of the thoracic nerve roots at the intervertebral foramen varies by location in the spine, with upper thoracic roots projecting upwards, middle thoracic roots oriented in a horizontal plane, and lower thoracic roots projecting downward. This variation influences the position of the dorsal root ganglion with regard to the spinal cord and the intervertebral foramen, with attendant variability in the extraforaminal extent of the dural sheath.

There are various foraminal ligaments closely related to the exiting nerve root that may be present in the thoracic spine intervertebral foramen [17]. The ligamentous structures vary in width and thickness from 2 to 5 mm and substantially decrease the aperture of the intervertebral foramen—while no data is reported specific to the thoracic spine, the actual aperture size in thoracolumbar spine among 49 human nonpathological intervertebral foramen was on average 31.5% less than the size of the osseous foramen [18]. The *superior* and *inferior corporopedicular ligaments* attach to the superior and inferior pedicle, respectively, and traverse obliquely anteriorly to the posterolateral vertebral body and associated annulus fibrosus. The *superior transforaminal ligament* attaches from the anterior inferior vertebral notch on the superior pedicle to the articular capsule. The *mid-transforaminal ligament* attaches from annulus fibrosus and superior and inferior corpopedicular ligaments to the articular capsule. The *inferior transforaminal ligament* extends from the junction of the annulus fibrosus and the posterior vertebral body to the superior articular facet. Lastly, the *suspensor radial ligaments* extend radially from the nerve roots to the related ligamentous structures. When present, the foraminal ligamentous relationships to the thoracic nerve root are the following: inferior corporopedicular ligament (posterosuperior), superior transforaminal ligament (anterosuperior), ligamentum flavum (posterior), superior corporopedicular ligament (anterior), and mid-transforaminal ligament (inferior). These ligaments can sequester the intervertebral artery and vein away in fatty areolar tissue ventral to the exiting nerve root exiting from the foramen anterior to the superior corpopedicular ligament, inferior to the mid-transforaminal ligament, and superior to the inferior transforaminal ligament [17]. 

### 3.4. Pathoanatomy of Subarachnoid Pleural Fistula

The dura of an emerging thoracic spinal nerve can be injured in the vicinity of the intervertebral foramen during extra-pleural dissection, neural element dissection, excessive rib retraction, or antecedent or inadvertent rib fracture [19, 20]. Furthermore, traction on a tumor adherent to the nerve can also accidentally tear the dura. Chest wall resections commonly breach dural integrity by requiring multiple rhizotomies at costovertebral joints or ligation of the nerve root at the intervertebral foramen. When the procedure requires sacrifice of the nerve root, thoracic surgeons are advised to approach with caution by applying large hemoclips or non-absorbable sutures to the nerve before division. Dissection of adjacent and adherent tumor from around the nerve is necessary to allow successful ligation. Forceful rib retraction can avulse the nerve root and create a dural defect. At the end of the procedure, patients should be routinely ventilated with increased intrathoracic pressure to raise subarachnoid pressure and identify any leak prior to chest closure.

The volume of CSF in the adult human cranial vault is approximately 150 mL and it is produced at a rate of 500 mL per day. This formation follows a circadian rhythm and is split nearly between 60% at the choroid plexus tissue and 40% extrachoroidal interstitial fluid movement from the brain parenchyma [21]. Absorption of CSF through the intracranial and spinal arachnoid villi begins at average CSF pressure of 68 mm H_2_O and rises linearly up to 250 mm H_2_O, with equivalence of formation and absorption rates occurring at 112 mm H_2_O [22]. This variability is a homeostatic response to maintain normal intracranial pressure and CSF volume.

In the presence of concomitant defects in the dura and the parietal pleura, the cyclic changes in the negative intrapleural pressure during respiration become important in directing the flow of CSF. This unidirectional movement is from the positive-pressure subarachnoid space to the negative pressure pleural space where the fluid accumulates. The persistence of this pressure gradient has the effect of continuously drawing CSF into the pleural space, with the flow impeding spontaneous closure of the fistula. Furthermore, while the pleural space can normally remove fluid through its lymphatic stomas at a clearance rate nearly 30 times the fluid formation rate [23]; excessive CSF flowing through the SPF along with altered lymphatic drainage by the underlying cancer or the surgical interruption of mediastinal lymphatic vessels can lead to fluid accumulation.

Spine surgeons are advised, when possible, to preserve the parietal pleura during exposure of the thoracic spine [24]. Such iatrogenic SPF lesions are more common after anterior approaches to thoracic tumors, probably a consequence of the greater likelihood of the requisite simultaneous damage to both the meninges and the parietal pleura. Indeed, Hentschel and coworkers [5] describe a retrospective series of 770 patients in whom nine developed a SPF, for overall incidences of 2.4% and 0.23% following anterior and posterior approaches, respectively.

A CSF leak that is identified intraoperatively should be treated primarily because iatrogenic SPF are unlikely to heal spontaneously. Whenever possible, the surgeon should perform simple ligation with hemoclips to arrest further leakage and to prevent the untoward complications of a persistent SPF. There are few other reported techniques of addressing this complication through a thoracotomy, though they can be of great value to the thoracic surgeon instead of changing to a prone position for a laminectomy. While thoracoplasty is possible, it is technically demanding near the intervertebral foramen and may lead to more deleterious bleeding or further dural injury. Neurosurgical consultation should be sought to evaluate options of foraminal widening with direct dural closure, or simultaneous or staged posterior laminectomy if necessary to provide definitive treatment. Patch grafts of muscle [5], omentum, and fascia have been described to be successful, secured in place using sutures or fibrin glue [1, 25].

### 3.5. Symptomatology of Subarachnoid-Pleural Fistula

During the postoperative recovery, the presentation of such lesions can be through pulmonary morbidity of large transudative pleural effusions or through neurological morbidity of intracranial hypotension, pneumocephaly, or central nervous system infection. Chest radiography can suggest the diagnosis of SPF when there is presence or rapid accumulation of pleural fluid or mediastinal widening. The pleural cavity is normally able to absorb CSF with clinical evidence of this found in ventriculopleural shunts for hydrocephalus [26, 27], rendering the incidence of SPF to be likely underestimated. Accumulation of CSF in the thoracic cavity is the most common presentation and can be identified by chest radiography demonstrating unilateral or bilateral pleural effusions with the presence of *β*2-transferrin in the pleural fluid [28]. This marker occurs nearly exclusively in the CSF, arising from the *β*1-isoform by the action of neuraminidase and is consequently highly sensitive and specific for traumatic or perioperative leak [6, 29–31]. It has been rarely reported to be falsely negative in surgically-confirmed SPF [28, 32], and false positives will occur in individuals heterozygous for the *transferrin *gene [33]. The latter can be avoided by electrophoretic evaluation on both the analyte fluid and a serum control from the same patient. The pleural fluid is otherwise characteristically clear having low nucleated cell count, with total protein consistently less than 1.0 g/dL and a pleural fluid to serum glucose concentration ratio between 0.5 and 1.0 [34]. 

Patients with neurological symptoms most commonly present with headache, altered mental status, or declining level of consciousness after recent thoracotomy [7]. Headache may occur as a consequence of CSF hypovolemia, meningitis, or pneumocephalus; the latter two are among the most significant neurological complications of SPF [5, 7, 35]. Pneumocephalus may arise by the presence of pneumothorax after open thoracotomy or tube thoracostomy. While plain skull radiographs may demonstrate the presence of intracranial air, such techniques are becoming obsolete in deference to the superior anatomic detail provided by CT scans. Accumulation of intracranial air under tension is a neurosurgical emergency and may indicate ventricular drain placement for decompression. The most characteristic physical finding of this condition would be *bruit hydroaerique*, a splashing sound induced by a rapid change in head position [36]. Chadduck [37] and Ladehoff [38] describe a rare but morbid complication of remote cerebellar hemorrhage following spinal surgery, with a consensus that downward cerebellar displacement accompanies the relative CSF hypovolemia, leading to tension and occlusion on the superior cerebellar bridging veins with consequent venous infarction and hemorrhage.

### 3.6. Diagnosis and Management of Subarachnoid Pleural Fistula

Diagnosis of a suspected CSF leak after a chest operation is best confirmed by CT myelography or radionuclide cisternography. The anatomic detail provided by CT myelography can aid in planning for surgical correction, but it carries a high false-negative rate [39, 40]. Nuclear cisternograms with infusion of ^111^In-DTPA is more sensitive for detection of SPF [5, 41], though does not provide anatomic detail and must hence be combined with other imaging for preoperative planning.

Chest tube drainage of the initial pleural effusion can provide both diagnostic and symptomatic therapeutic benefit. However, it can be deleterious to if suction is applied because it will promote continuous egress of CSF into the pleural space and worsen CSF hypovolemia, while also maintaining patency of the SPF. Instead, a water-tight seal should be applied to allow gravity-dependent drainage of pleural fluid accumulation. Insertion of a lumbar drain can divert CSF flow away from the fistula, though it is rare that such maneuvers are successful, and Katz and coworkers [42] report about one patient in whom this leads to pneumonia and meningitis. Furthermore, lumbar drainage cannot control movement of air into the subarachnoid space in the setting of persistent air leak from the lung parenchyma after lobar or sublobar resection. Specific treatment of the fistula is dictated by the defect size and progression of the patient's symptoms, with larger fistulae often requiring surgical closure, with one or two level posterior laminectomy preferable to reopening of the thoracotomy for securing repair of the defect. Reported maneuvers include primary surgical closure with reinforcement by application of omentum, muscle [5], or fat [1] patches to facilitate repair. Other materials that have been used include a methylmethacrylate plug wrapped in pleura [43], autologous blood patch [44], or thrombin soaked gelatin [45].

## 4. Conclusion

Iatrogenic SPF following resection of thoracic tumors is a rare complication that introduces significant perioperative morbidity to the affected patient as a consequence of pulmonary or neurological symptoms. The seriousness of this complication should not be underestimated as there may be life-threatening consequences of tension pneumocephalus, cerebellar infarction and bleeding, meningitis, or massive pleural effusion. The thoracic surgeon operating in the vicinity of the intervertebral foramen must be familiar with the regional and applied anatomy at this site, including thorough knowledge of osseous, ligamentous, neural, and vascular structures. This can afford careful avoidance of dural injury and may help identify sites of CSF leakage when such injury has occurred to facilitate immediate repair.
